# Interaction of Motility, Directional Sensing, and Polarity Modules Recreates the Behaviors of Chemotaxing Cells

**DOI:** 10.1371/journal.pcbi.1003122

**Published:** 2013-07-04

**Authors:** Changji Shi, Chuan-Hsiang Huang, Peter N. Devreotes, Pablo A. Iglesias

**Affiliations:** 1 Department of Electrical and Computer Engineering, Whiting School of Engineering, Johns Hopkins University, Baltimore, Maryland, United States of America; 2 Department of Cell Biology, School of Medicine, Johns Hopkins University, Baltimore, Maryland, United States of America; 3 Biological Physics, Max Planck Institute for the Physics of Complex Systems, Dresden, Germany; North Carolina State University, United States of America

## Abstract

Chemotaxis involves the coordinated action of separable but interrelated processes: motility, gradient sensing, and polarization. We have hypothesized that these are mediated by separate modules that account for these processes individually and that, when combined, recreate most of the behaviors of chemotactic cells. Here, we describe a mathematical model where the modules are implemented in terms of reaction-diffusion equations. Migration and the accompanying changes in cellular morphology are demonstrated in simulations using a mechanical model of the cell cortex implemented in the level set framework. The central module is an excitable network that accounts for random migration. The response to combinations of uniform stimuli and gradients is mediated by a local excitation, global inhibition module that biases the direction in which excitability is directed. A polarization module linked to the excitable network through the cytoskeleton allows unstimulated cells to move persistently and, for cells in gradients, to gradually acquire distinct sensitivity between front and back. Finally, by varying the strengths of various feedback loops in the model we obtain cellular behaviors that mirror those of genetically altered cell lines.

## Author Summary

Chemotaxis is the movement of cells in response to spatial gradients of chemical cues. While single-celled organisms rely on sensing and responding to chemical gradients to search for nutrients, chemotaxis is also an essential component of the mammalian immune system. However, chemotaxis can also be deleterious, since chemotactic tumor cells can lead to metastasis. Due to its importance, understanding the process by which cells sense and respond to chemical gradients has attracted considerable interest. Moreover, because of the complexity of chemotactic signaling, which includes multiple feedback loops and redundant pathways, this has been a research area in which computational models have had a significant impact in understanding experimental findings. Here, we propose a modular description of the signaling network that regulates chemotaxis. The modules describe different processes that are observed in chemotactic cells. In addition to accounting for these behaviors individually, we show that the overall system recreates many features of the directed motion of migrating cells. The signaling described by our modules is implemented as a series of equations, whereas movement and the accompanying cellular deformations are simulated using a mechanical model of the cell and implemented using level set methods, a method that allows simulations of cells as they change morphology.

## Introduction

Cells have a remarkable ability to sense the direction of chemical gradients and respond by polarizing and migrating toward attractants. Chemotaxis is one of the fundamental properties of single cell organisms, such as bacteria and amoebae, as well as multicellular systems. Experiments suggest that chemotaxis involves the coordinated action of separable but interrelated processes: motility, gradient sensing, and polarization [1]. In fast moving amoeboid cells, such as the social amoeba *Dictyostelium discoideum* or human neutrophils, motility arises from the periodic extension of actin-rich pseudopods whose nature is quite similar in chemoattractant stimulated and unstimulated cells. Gradient sensing refers to the cell's ability to interpret extracellular gradients and to respond by directing intracellular proteins to the site of highest chemoattractant concentration. Experiments in eukaryotic cells in which motility has been impaired by inhibitors of actin polymerization, such as Latrunculin, demonstrate that gradient sensing occurs even in immobile cells, indicating that cells employ a spatial sensing mechanism that does not depend on movement. Finally, polarization is the propensity of cells to assume stable anterior and posterior edges leading to an elongated morphology. The anterior region is more sensitive to chemoattractants so that in response to a changing gradient polarized cells turn towards the new direction [2]. In contrast to gradient sensing, polarization depends on intact cytoskeleton.

The study of chemotaxis has benefitted greatly from the interplay between experimental and theoretical studies [1], [3]–[5]. A number of recent models propose that chemotaxis is a consequence of an excitable network whose activity is biased in the direction of chemoattractant stimuli [6]–[10]. Motility can be achieved if this activity directs pseudopodial protrusions [11], [12]. The basis for these models is the observation that cytoskeletal and signaling pathways in cells exhibit excitable behavior, in the form of patches and waves of activity seen along the cell cortex, and that these activities coincide with the location of protrusions [10], [13]–[21].

We start with a previously described local excitation, global inhibition biased excitable network (LEGI-BEN) that captured some of the experimentally observed features of spontaneous and chemoattractant-induced signaling events, and add two modules. First, we use level set methods [22], [23] and a viscoelastic mechanical model to simulate cytoskeleton-mediated cellular deformations and movements. Second, we incorporate a cytoskeleton-dependent polarity module that confers both the persistent migration seen in unstimulated cells as well as other characteristics of polarized cells. We use the model to consider a number of *in silico* mutants and use the resulting simulated results to consider the possible biochemical identities of elements in the model. Whereas various previous models can account for different subsets of behaviors of chemotaxing cells, we show that the complete modular framework of the polarized LEGI-BEN model presented here accounts for nearly all the reported observations.

## Results

### Linking the Activity of the Excitable Network to Cellular Protrusions

**Figure 1:**
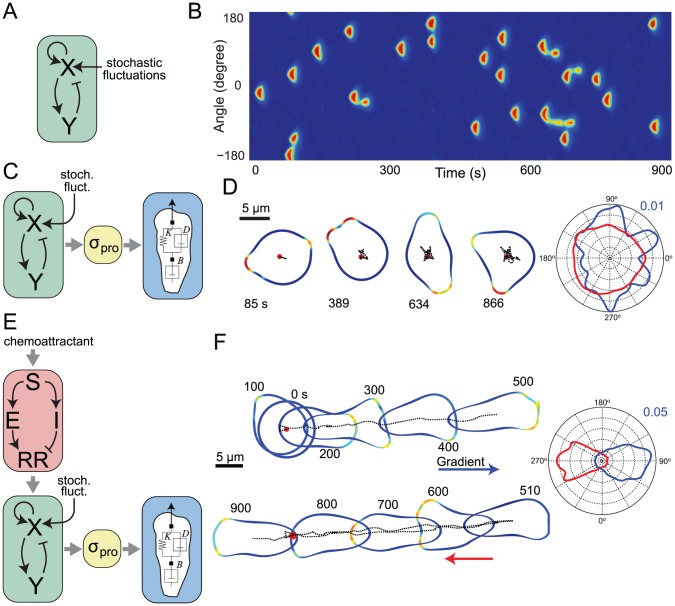
The LEGI-biased excitable network model (LEGI-BEN). (A) The excitable network module is implemented as an activator (*X*)-inhibitor (*Y*) system that is triggered by stochastic fluctuations. (B) Kymograph of a one-dimensional simulation in the absence of chemoattractant stimulus. The colors refer to activity of *Y* (plots of *X* show similar, though noisier, behavior) around a cell. Blue indicates low activity; red marks high activity. (C) Coupling of the excitable network to protrusive stresses (σ_pro_). Our simulations assume that the protrusive stress is proportional to *Y*. The cell's mechanical behavior is described by the viscoelastic model shown. (D) Level set simulations in which protrusive stresses coincide with the location of high activity drive cellular deformations. The colors around the membrane are the same as in panel B. The dotted lines trace the trajectory of the cell centroid (starting point is the red circle). The directional history of activity for this sample simulation is shown in the radial plot on the right in blue. The red line represents the average activities of 20 simulations lasting 900 seconds (Video S1). (E) The local excitation (*E*)-global inhibition (*I*) module (LEGI) takes chemoattractant stimulus (*S*) and drives a response regulator (*RR*). Response regulator acts to bias the activity of the excitable network. (F) Level set simulations of the cell migrating in response to changing chemoattractant gradients. A gradient was applied at time 180 s at 90° and moved at time 500 to 270°. (Note that, to avoid the cell shapes being superimposed, we have moved the trajectory during the second half of the simulation below that of the first; the dotted lines show how the two halves of the trajectory overlap.) The radial plot shows the average activities in response to the two gradients. The blue line is for the time period from 0 s to 500 s; red is from 500 s to 900 s (Video S2).

Previously we and others have proposed that the spontaneous patches of signaling activity seen in motile cells could be explained by an excitable network (EN) consisting of two components: an activator (*X*) and an inhibitor (*Y*) (Fig. 1A) [7]–[10], [15]. The activator, which involves an autocatalytic positive feedback loop, drives the inhibitor, which provides negative feedback. Experimentally, excitable behavior is observed in cells that are not stimulated by chemoattractant, indicating that the chemoattractant receptor is upstream of and not part of the EN [18]. The spontaneous nature of these activities can be recreated by including a stochastic element that triggers the EN randomly. The activity of the EN around the perimeter of a one-dimensional model of the cell can be observed by plotting the level of the activator or inhibitor as a kymograph (Fig. 1B). These patches represent localized signaling events that drive cell protrusion.

To determine whether this model could recreate cellular protrusions leading to random cell motility, we coupled the EN to a mechanical module of the cell cortex implemented in the level set framework (Fig. 1C, Methods.) (Computational methods that account for changes in cell morphology during migration are reviewed in Ref. [5]). The mechanical description of the cell was identified based on micropipette aspiration experiments using *Dictyostelium* cells [22]. The mechanical module incorporates several passive stresses, including the effect of cortical tension driving Laplace-like pressures on the cell and volume conservation. It also includes active stresses allowing us to test the effectiveness of the EN in driving cellular motion. In our simulations, the activity of the EN was coupled to protrusive forces, so that higher activity at one location gave stronger protrusive stress (Fig. 1C, D). We envision that the effects of the EN on the cytoskeleton mediate these protrusive forces. In these simulations, local protrusions appeared randomly around the cell, as would be expected in a cell undergoing random motility (Fig. 1D; Video S1). Analysis of these simulations over time revealed that the activities were uniformly distributed at the population level over long time scales, though localized fluctuations do occur in shorter time scales (Fig. 1D; inset). The localized forces caused by these heterogeneities, however, were not sufficiently persistent to propel the cells in a meaningful way. Thus, the cellular boundary extended in random directions, but the migration rate of the cell was negligible.

### Incorporation of a Directional Sensing Module

To test the effect of a chemoattractant gradient we incorporated a local excitation-global inhibition (LEGI) mechanism to the excitable network, creating a LEGI-BEN system (Fig. 1E), as previously described [8], [10]. Because the response regulator in the LEGI mechanism increases at the front and decreases at the back, it brings the excitable system closer or farther from the threshold at the front and back, respectively. This biases the likelihood of triggering activity in response to external chemoattractant signals. When a gradient was applied to an unstimulated cell, the activity of the cell increased everywhere around its perimeter (Video S2). Thereafter, signaling activity was found preferentially at the side of the cell experiencing the greatest chemoattractant concentration (Fig. 1F; Video S2). The cellular response to a change in gradient was nearly immediate, and this was true for both steep (19%) and shallower (6%, not shown) gradients. When simulating cell shape changes elicited by this gradient, we found that after the application of the stimulus, the cell elongated and moved in the direction of the gradient. Analysis of the activity showed that the directional response is quite accurate and most of the activities are within the −30° to 30° region relative to the direction of gradient (Fig. 1F, inset). The magnitude of the protrusive force was chosen so that, at steady-state, the cells moved at approximately 10 µm/min. After a shift in the direction of the gradient, the cell stopped and reversed direction nearly instantly (Fig. 1F; Video S2).

**Figure 2:**
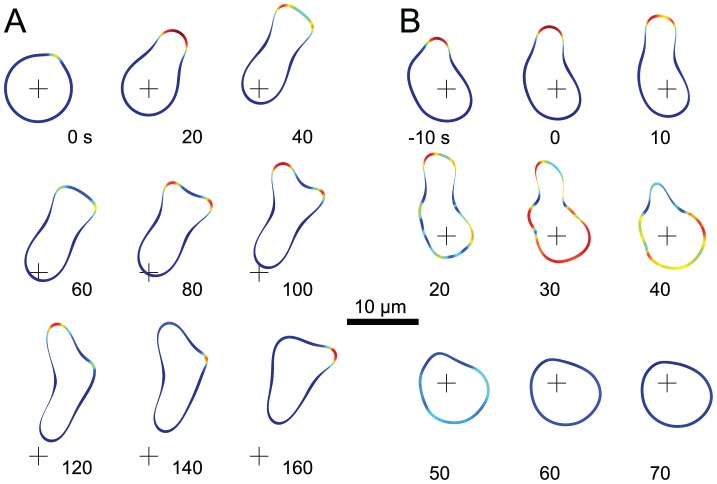
Changes in morphology of motile cells. (A) The signaling activity and corresponding cellular morphology is shown for a migrating cell in a gradient. This cell demonstrates pseudopod splitting, pseudopod retractions, and a zig-zag pattern of activity. The cross is placed for spatial reference. (B) This cell experienced a global (spatially uniform) chemoattractant stimulus at 0 s. The ensuing period of high activity (30 s) causes the cell to start rounding up; this rounding increases during the refractory period of the excitable network (70 s).

The LEGI-BEN coupled to the mechanical module recapitulates several consistently observed cellular behaviors such as “pseudopod splitting” and “cringing.” First, cells often generate a new pseudopod by splitting an existing one [24]–[26]. In chemotaxing cells, these bifurcations appear as a series of left-right extensions. Our simulations of chemotactic cells also exhibited “pseudopod splitting” (Fig. 2A). Because the midpoint of the responding area has the highest activity, negative feedback shuts down this region first, the signals propagate away in opposite directions and the pseudopod splits. Though more pronounced in stimulated cells, it was also observed during spontaneous movement (Fig. 1D, 389 s). Nascent extensions grew from localized patches of high signaling activity. These pushed the cell forward, but eventually split in two (Fig. 2A, 20–40 s). While these extensions sometimes co-existed for a while (Fig. 2A, 80 s), one usually won out, at which point the “losing” pseudopod appeared to retract into the cell (Fig. 2A, 100–120 s). This pattern often repeated itself, giving rise to the appearance of side-to-side strokes propelling the cell.

Application of a spatially uniform dose of chemoattractant to a *Dictyostelium* cell results in a series of changes in cell morphology. Cells stop moving and then transiently contract (or cringe). This is followed by spreading and eventual resumption of movement. Our simulation also recreated this phenomenon (Fig. 2B). Approximately 30 s after stimulation, the cell experienced a mostly global rise in signaling activity (Fig. 2B). At this point the protrusive stresses in our model sought to push out the cell everywhere, but because of the passive constraints on cell morphology, this global increase in activity had the effect of rounding up the cell. The increase of activator subsequently generates more inhibitor. Once the inhibitor prevails, it suppresses activity all around cell. Thus, the global firing was followed by an absence of signaling caused by the refractory period that follows the firing of an excitable network leading to further rounding of the cell. This was followed by spreading and eventually activities reappeared stochastically around the parameter (not shown).

### Incorporation of a Polarity Module

While the simulations of Fig. 1 accurately displayed the chemotactic behavior of unpolarized cells, they lacked two important characteristics observed in stably polarized cells. First, polarized cells moving in the absence of chemoattractants travel in a persistent random walk, and this persistence is a result of having pseudopodia extend in the same direction [26]–[31]. Second, they have an elongated morphology with activity confined to the anterior portion of the cell. *Dictyostelium* cells at an early stage in their developmental program are mostly unpolarized but the degree of polarization increases as they differentiate [32]. In contrast, once activated, neutrophils are highly polarized.

**Figure 3:**
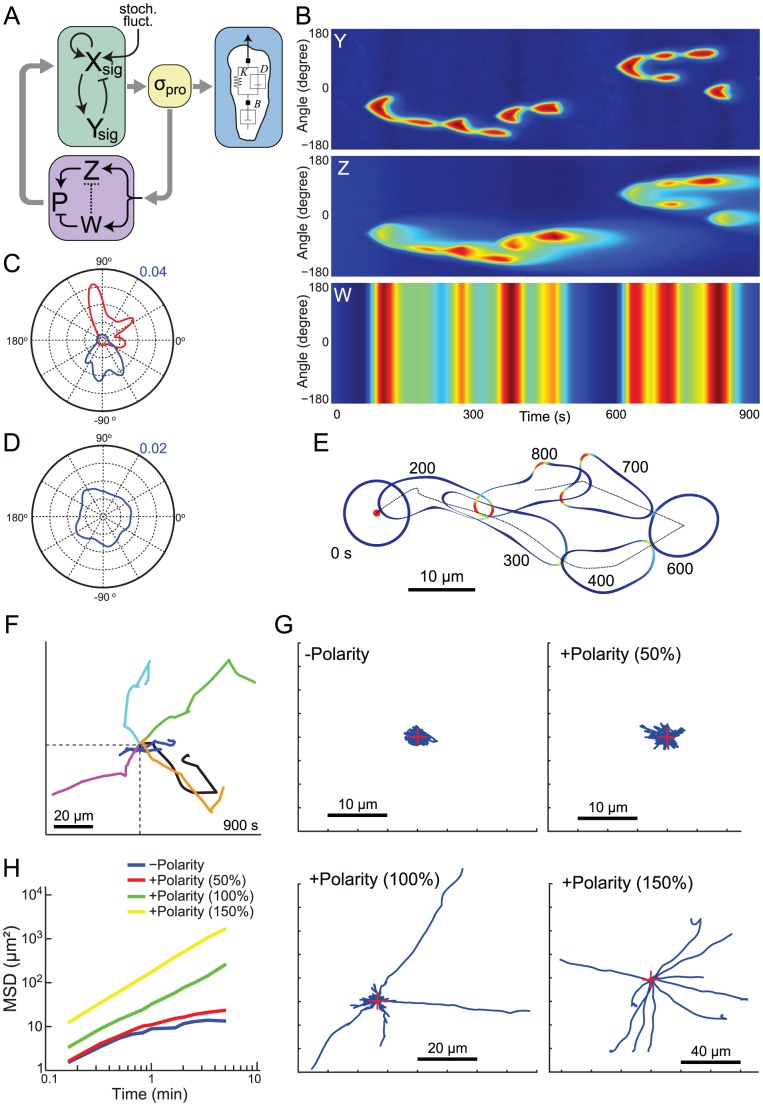
Polarized-biased excitable network in unstimulated cells. (A) Excitable network with polarization mechanism, which consists of complementary local positive (*Z*) and global negative (*W*) feedback loops. The inhibitory term (*W*) can work either directly on polarity (*P*) or by inhibiting *Z* (dotted line). The latter could represent depletion. In simulations we assumed the former. (B) Simulation results of excitable network with polarity. Kymographs show the spatio-temporal distribution of *Y*, *Z* and *W*. (C) Analysis of activity for the simulation of panel B. The blue and red lines represent the activities (*Y*) along the perimeter during the periods 70–490 s and 600–850 s, respectively. (D) Average activity of 40 simulations, each 900 s long. (E) Level set simulation of unstimulated cell shows persistent movement in the absence of stimulus. (F) Centroid trajectories of six different cells during 900 s (Video S3). The asterisk denotes the trajectory of the cell from panel E (the trajectory in panel E was rotated for better presentation). (G) Centroids of unstimulated cells with varying strengths of the polarity module's contribution during 600 s simulations (n = 10 each). (H) Average mean-square displacements as a function of time for the simulations of panel G.

To overcome these limitations and recreate more realistic cellular behavior, we introduced an additional polarity module to create a polarized-EN system. To achieve polarity we incorporated in our model a secondary set of feedback loops from the cytoskeleton indicated by an arrow linking protrusive stress to the signaling element (Fig. 3A). Positive feedback loops have been a feature of most models of polarization (reviewed in Refs. [4] and [33]) based on experimental evidence that polarization is a consequence of such loops between actin and signaling proteins (e.g. [34]). In our context, a local positive feedback loop (element *Z* in Fig. 3A) biases the likelihood of subsequent activity at the location of high protrusive stresses. Thus, because stresses are caused by localized increases in signaling activity, whenever high activity occurs at one location, it is more likely that subsequent bursts of activity will occur at that position again. However, only adding a positive loop is not enough to realize polarity. First, there is a lifetime to this persistence, and so the contribution of this loop is expected to subside. Second, without a counteracting negative feedback, the effect of the loop could increase over time throughout the cell, and so lead to hyperactive cells. We therefore included a global negative feedback loop that reduces the activity throughout the cell. This loop was implemented as a separate component (element *W* in Fig. 3A) that acts to reduce polarization. A second possibility for this inhibition would be to act against *Z* directly (dotted line in Fig. 3A) as might be expected if the inhibition were in the form of substrate depletion. Negative feedback loops are less common in models of polarization, though several models assume mass conservation of the polarity element, which has the same net effect [35]–[37]. Importantly, averaged over the surface of the cell, the two loops cancel each other out. However, the net effect of the two components is positive at locations of high stress and is negative elsewhere.

Simulations of the polarized-EN system in an unstimulated cell showed persistence in the location of the excitable behavior (Fig. 3). For example, in the kymographs of Fig. 3B, high activity during the period 0–500 s was centered around −90° whereas, after 600 s, it was around +90° (Fig. 3C). These kymographs show that polarity had a localized and transient biasing effect in terms of activity. However, integrating the activity of 40 simulations (each 900 s long) showed that, on average, the location of high activity was uniformly distributed around the cell as would be expected in a randomly migrating cell (Fig. 3D). To examine the temporal effect of the polarization module on the appearance of excitable behavior in any one direction, we computed the autocorrelation function for the activity at fixed angles (Fig. S1). Without the polarity module, the autocorrelation decreases to 0.3 in approximately 30 seconds and approaches zero after about two minutes. With the polarity module, it plateaus at about 0.4 after 30 seconds. The level of this plateau can be changed by varying the coefficient that controls the lifetime of the polarization element. To observe the effect of this persistence on cell motility, we used the polarized EN (Fig. 3A) to simulate cell motility and changes in cell morphology (Fig. 3E). These simulations showed that unstimulated cells could move significant distances, though the direction and net velocity were random (Fig. 3F; Video S3). Moreover, as the strength of the polarization increased (by varying parameter φ), the cells drifted farther away from the initial position, as measured by the mean-squared displacement (Fig. 3G,H). These results follow closely observations which show that randomly migrating *Dictyostelium* cells 5.5 hours into development have mean-squared displacements that are approximately ten times higher than newly developed cells [31]. The length of development time also correlates with the degree of morphological polarization [32].

### Integration of the Polarization Module with the LEGI-BEN

#### Response to uniform stimuli and gradients

**Figure 4:**
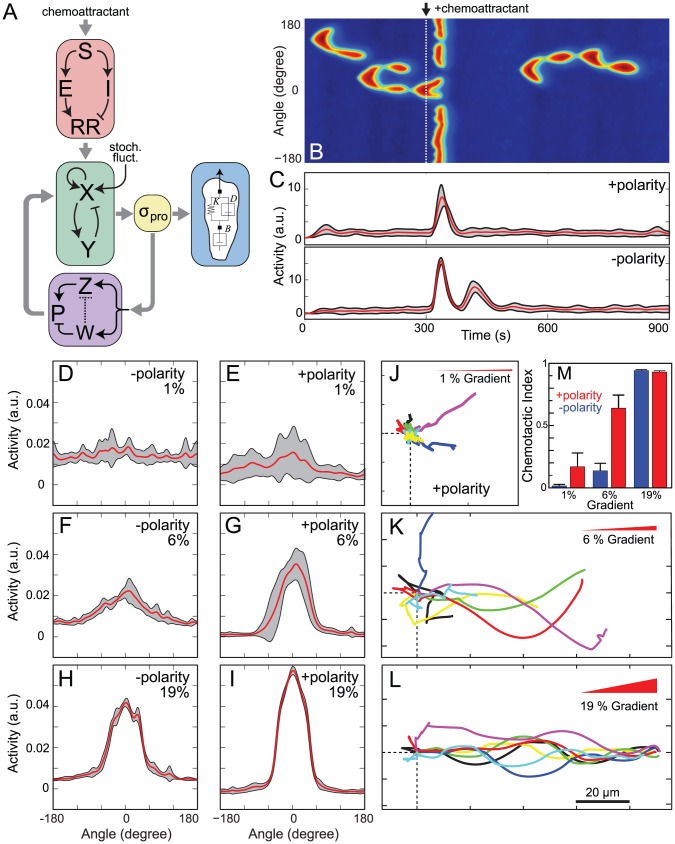
Response of polarized cells to external stimuli. (A) LEGI-Biased excitable network with polarity. (B) Signaling activity (*Y*) around the cell in response to a spatially uniform stimulus at 300 s. (C) Average activity (red) and variance (one standard deviation) for 20 simulations. Activities are integrated around the whole cell perimeter. Top graph represents the results of the polarized, LEGI-BEN; bottom is for the model without polarity. (D–I) Radial distribution of signaling activities of the model responding to 1% (D,E) or 6% (F,G) and 19% (H,I) gradients. Panels D, F and H are for cells without the polarization module; panels E, G, and I include this module. Data are average for ten simulations of 900 s. Red lines denote mean value and black lines represent one standard deviation. (J–L) Sample trajectories of the cell centroid for level set simulations of cells incorporating the polarization module under various gradients (all pointing to the right). The dotted lines point to the starting point and lines represent individual cells' trajectories. All the simulations were run for 900 seconds. (M). Chemotactic indices for simulations of cells migrating under various gradients with or without the polarization module. Error bars denote one standard deviation based on seven simulations each.

We next considered the effect of chemoattractant stimuli on our polarized-EN, by reintroducing the LEGI mechanism (Fig. 4A). We refer to this complete model as polarized LEGI-BEN. We first simulated the response to a spatially homogeneous stimulus. Before stimulation, the cell displayed random spontaneous activity (Fig. 4B, C). In response to the stimulus, activity increased transiently around the perimeter, lasting approximately 30 seconds. Thereafter, activity subsided throughout, before resuming their spontaneous activity (as the LEGI mechanism adapted to the uniform stimulus). In contrast to cells lacking the polarity mechanism (Fig. 4C, bottom), which displayed a strong secondary peak of elevated activity around 120 seconds after the chemoattractant stimulus, cells with the polarity mechanism do not exhibit this secondary peak (Fig. 4C, top). Consistent with our simulation results, there is widespread experimental evidence for a second peak in early-stage less polarized cells, but it is less pronounced (or nearly absent) in well-developed polarized cells [32]. Without the polarity module, the second peak appears because the LEGI module has not adapted completely, and so secondary firings of the excitable system take place. With the polarization module, the presence of an extra negative feedback loop (*W*) makes these less likely, effectively eliminating them.

We next tested the effect of gradients of varying steepness (Fig. 4E–M). In all cases the activity of the cell aligned preferentially in the direction of the gradient. In 19% gradients the activity was concentrated in an arch around ±30° and lateral pseudopods were rarely observed. In 6% gradients the response was still predominantly biased in the direction of the stimulus, but lateral pseudopods were observed occasionally. In 1% gradients there was alignment, but considerable more spread.

The alignment of the activity with the gradient in simulations of cells lacking the polarity mechanism also showed dependence on the gradient steepness (Fig. 4D, F, H). In all cases, the activity in polarized cells showed better alignment with the gradient and less variability. Using the level set simulations to compare the trajectory of cells in response to these varying gradients revealed a similar gradient-dependency. Cells responded better to the steeper gradients, as evidenced by straighter trajectories (Fig. 4J–L) and greater chemotactic indices (CI) (Fig. 4M). For cells with the polarity module, these ranged between 0.17±0.02 to 0.64±0.21 to 0.93±0.02 in 1%, 6% and 19% gradients, respectively (*n* = 7 in each case). These are similar to reported values in the literature. For example, CIs of 0.2, 0.6 and 0.9 were measured for cells chemotaxing in relative gradients of 1.4%, 4.8% and 10%, respectively [26]. The latter gradients were imposed by a cAMP-filled micropipette. In gradients created by microfluidics, which are closer to ours since they are not formed by a point source, CIs of 0.1–0.3, 0.15–0.4 and 0.96–0.99 have been measured in 1.25%, 2.5% [38] and13.2% gradients [39].

#### Response to shifts in gradient

**Figure 5:**
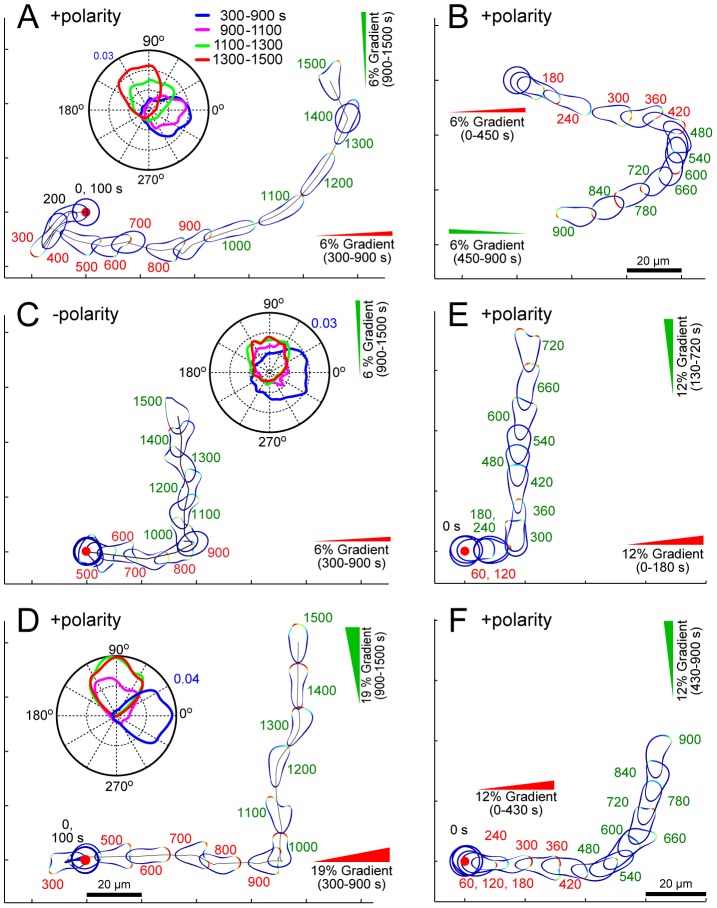
Polarized cell's response to changes in the direction of the gradient. (A–D) In these simulations the arrows indicate the direction of the gradient from 300–900 s (red) and from 900–1500 s (green). Cells were unstimulated from 0–300 s. The simulations differ as to the steepness of the gradient: 6% (A–C) and 19% (D), whether the polarity module is active (A, B, D) or not (C), and the direction of the second gradient: 90° (A,C,D) and 180° (B). The insets show the direction of the signaling activity relative to the cell for various time intervals. See also Videos S4, S5, S6. (E, F). These simulations show the response of a cell with all its modules (Fig. 4A) responding to a change in the direction of a 12% gradient for which the interval during which the first gradient is imposed varies from 130 s (E) to 430 s (F). See also Videos S7 and S8.

One of the main differences between polarized and unpolarized cells is in the response to changing gradients [2], [40]. In a cell with the polarity module, we first applied a 6% gradient, maintained this for 10 minutes, and then shifted the gradient 90° (Fig. 5A; Video S4). Prior to any stimulus, the cell migrated randomly. After sensing the first gradient, the cell slowly aligned itself in the direction of the gradient and began migrating (Fig. 5A). After the direction of the gradient changed, the cell maintained its axis of activity and began a slow turning motion eventually realigning with the new gradient (Fig. 5A). A similar turning motion was observed when the direction of the 6% gradient was changed 180° (Fig. 5B).

We next repeated this simulation in a cell without the polarity mechanism (Fig. 5C; Video S5). The response in the direction of the initial gradient was similar, although unpolarized cells lined up faster than polarized cells. Furthermore, after the change in the direction of the 6% gradient, the cell immediately shifted its activity in the new direction, no longer extending pseudopods in the old front but instead focusing its activity in the direction of the new gradient (Fig. 5C inset). Thus, the cell trajectory exhibited a nearly 90° turn. Finally, we carried out this simulation in a cell with the polarity module, but in the presence of 19% gradients (Fig. 5D; Video S6). The response to the initial gradient was similar to the previous simulations, though the activity in response to the steeper gradient was more focused than that toward the shallower gradient (as previously observed in Fig. 4C, D) enabling the cell to move further along the gradient during the initial 900 s (compare the location of the cells at 900 s in Fig. 5A and 5D). After the change in gradient location, however, the polarized cell made a sharp 90° turn towards the new gradient. Thus, the response of a polarized cell to steep (19%) gradient changes was similar to that of an unpolarized cell to shallower (6%) gradient changes. These simulations show that polarity can be overcome by sufficiently strong gradients.

It has been observed experimentally that polarity can also be reinforced by a period of directed movement in a gradient [41]. To investigate how the time during which a cell is exposed to a gradient affects the development of polarity, we carried out simulations in which the time between application of the two gradients was altered. In Fig. 5E, F, cells migrated in response to a 12% gradient. The location of this gradient was changed 90° after either 130 (Fig. 5E; Video S7) or 430 s (Fig. 5F; Video S8). When the initial migration time was small, the cell made a sharp turn, displaying little polarity. However, when the cell had been migrating longer in the gradient, the cell displayed the turning behavior associated with polarized cells. These simulations show that, in our model, as in real cells, polarization is a property that develops over time, and is reinforced by the time during which the cell is exposed to a stable gradient.

#### Response to multiple gradients

**Figure 6:**
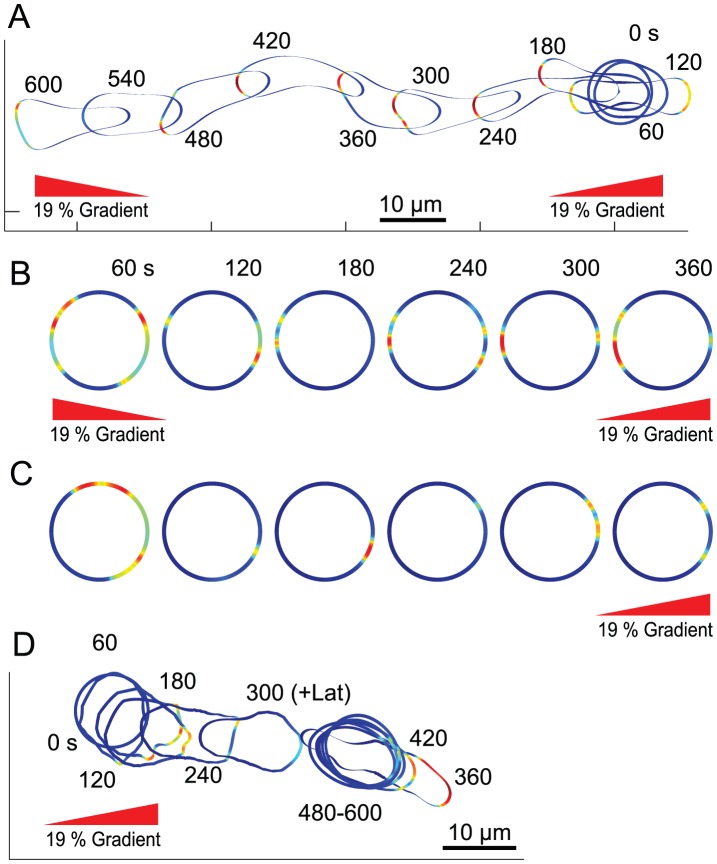
Effect of conflicting gradients. (A, B) In these simulations, two 19% gradients were applied 180 degrees apart. Panel A shows the response of a cell with all components in Fig. 4A (Video S9); Panel B is that of a cell lacking motility and polarization (Video S11). Video S10 shows the response of a motile cell lacking the polarity module. (C) Response of an immobile cell to a single 19% gradient (Video 12). (D) Level set simulation of cell migration under gradient (applied at 0 s) with Latrunculin treatment at 300 s. The total simulation time is 600 s.

When confronted by conflicting gradients, unpolarized, immobile cells (e.g. Latrunculin-treated) show elevated levels of signaling activity in the direction of both sources [42], a response that is recreated by the LEGI mechanism on its own [43]. Here we simulated the effect of conflicting gradients on the complete model of the cell. We started with a circular, unstimulated cell, applied two 19% gradients 180° apart and maintained these gradients no matter where the cell moved (Fig. 6A; Video S9). At first, the cell sometimes hesitated and, in some cases, even tried to extend pseudopods in both directions (e.g. at 120 s). However, as the cell polarized, one direction won out and the cell migrated in this direction. In contrast, cells that lacked the polarity module oscillated but never settled on either source (Video S10). Thus, polarization enables cells to select between two competing sources [3], [44]. We also simulated the effect of Latrunculin treatment by setting the cytoskeletal link to the mechanical module to zero irrespective of the EN behavior. The simulated Latrunculin-treated cells displayed activity in both directions throughout the period of the simulation (Fig. 6B; Video S11). Interestingly, however, the stochastic component in the signaling meant that, while the activity peaks pointed towards both gradients “on average,” the relative strengths varied over time. This stochastic behavior was also observed in cells that were stimulated with only one gradient (Fig. 6C; Video S12). We also simulated cells that were initially moving in response to an external gradient and to which Latrunculin was added, by gradually reducing the link to the mechanical module. These cells rounded up though they continued to signal in the direction of the gradient (Fig. 6D).

### Generation of “Mutant” Behavior by Altering Model Parameters

**Figure 7:**
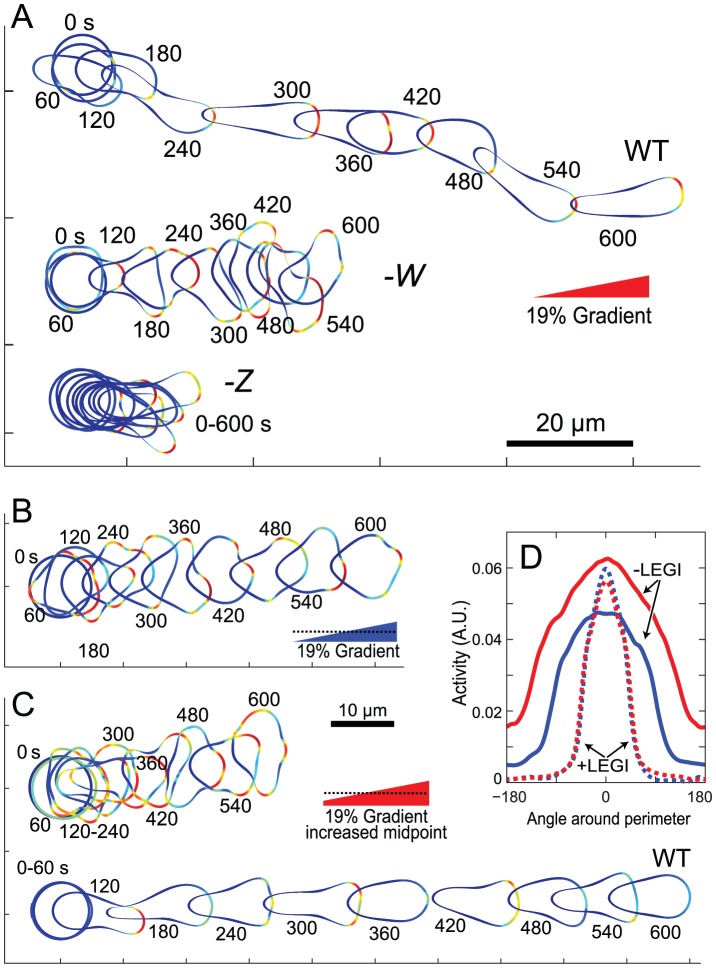
Response of cells with altered modules. (A) Response to a chemoattractant gradient of cells with reduction in feedback strengths in *W* or *Z* by 50%, compared to WT cells (Video S13). (B, C) Response of a cell to a gradient in which the LEGI inhibitor is not regulated by receptor occupancy assuming that the midpoint in gradient concentration is changed (Videos S14 and S15). The bottom cell in panel C shows the response of a WT cell in response to the increased midpoint concentration. (D) Directional distribution of the responses from panels B (blue) and C (red) with different gradient midpoints. Dotted lines show the corresponding distributions for the model with the LEGI module.

We next considered the effect of altering the strengths of individual loops in the signaling network. We first reduced the strength of the negative feedback loop in the polarization module by 50%. These cells could sense the gradient, however their signaling response, though still pointing on average in the direction of the chemoattractant gradient, was considerably broader (Fig. 7A, top and middle cells; Video S13). This resulted in chemotaxing cells that had multiple simultaneous protrusions which, in many cases, did not point directly towards the source. The cell morphology was quite different from the WT cells, with a broad area facing the gradient. The net movement was also slower. Cells where the negative feedback loop in the excitable network was reduced showed similar patterns of activity [10]. We also investigated the effect of diminishing the strength of the positive feedback loop (through *Z*) by 50%. The signaling in these cells was aligned with the external gradient. However, the overall level of activity was lower and so the cells moved only slowly in the direction of the gradient (Fig. 7A).

Lastly, we considered the role of the LEGI mechanism in enabling directional migration. We applied gradients to the cell in which the LEGI inhibitor was not regulated by receptor occupancy but is instead kept constant at the basal level. This prevents the LEGI mechanism from adapting to spatially uniform stimuli, although chemoattractant gradients are still sensed and pass on the directional signal to the excitable network. These cells could migrate in the direction of the gradient, though the effectiveness was significantly impaired (Fig. 7B; Video S14). When we raised the midpoint of the chemoattractant signal, as might be expected when cells approach a chemoattractant source, the chemotactic efficiency was further impaired compared to WT cells (Fig. 7C; Video S15). Comparing the activity of the EN in both situations (Fig. 7D) shows that the lack of adaptation causes the level of activity to rise throughout the cell perimeter, and this has a negative effect on movement, as multiple pseudopods can occur simultaneously and in the wrong direction. This was not the case for cells with an intact LEGI mechanism, where the inhibitor “filters out” the mean level of chemoattractant (Fig. 7D).

## Discussion

### Rationale for the Framework of Interacting Modules

Chemotactic cells display a variety of behaviors under various experimental conditions (Table 1). 1) Migrating cells display persistence. 2) New pseudopodia appear to split from previous ones. 3) The pseudopodia that bring about random migration coincide with patches of elevated signaling as well as cytoskeletal activity. 4) The cytoskeletal and signaling activities propagate as waves which lead to the patches of activity seen on the pseudopodia. The propagating waves suggest that these networks are excitable. 5) When exposed to spatially uniform chemotactic stimuli, cells “freeze” movement and then round up or “cringe”, then spread projections in multiple directions, and finally resume normal migratory behavior. 6) These events are driven by a stereotypical kinetically complex signaling response (i.e. Ras activation or PIP_3_ production) which is observed in immobilized as well as control cells. Within seconds of stimulus addition, cells produce an initial response around the whole perimeter that shuts off rapidly within 30 seconds and is followed by secondary patches lasting several minutes. 7) During persistent stimulation cells eventually adapt to the current level of stimulation but will respond again if the stimulus is increased or is reapplied after a period of recovery. 8) When cells are exposed to a gradient of chemoattractant, they produce directional responses and migrate directionally. 9) In immobilized cells, patches of response are stochastic but biased towards the high side of the gradient. 10) The directional response is amplified compared to the external gradient in the sense that it is confined to the anterior of the cell. 11) Immobilized cells exposed to two gradients produce responses on both ends, while migrating polarized cells choose one or the other sources. 12) Adaptation enables chemotactic cells to adjust their sensitivity and respond only to the steepness but not the midpoint concentration of the gradient. 13) The intrinsic polarity of cells is regulated. In *Dictyostelium*, for example, developed cells are more polarized than young cells. Polarity can also be enhanced by a period of migration in a gradient. 14) Polarized cells will turn when the gradient is shifted rather than creating a new front. As shown in Table 1, the modular framework of the polarized LEGI-BEN model accommodates all of these behaviors and experimental conditions whereas earlier models only account for various subsets of them.

**Table 1:** Behaviors simulated.

Behavior [Ref.]		Fig.	A	B	C	D	E	F	G
***Unstimulated cells***									
**1)**	Persistent motion [26]–[31].	3B–H	+	−	−	−	NA	−	+
**2)**	Pseudopod splitting [24]–[26]	2A	+	−	−	−	NA	−	+
**3)**	Random patches [63]–[65]	1D	+	+	+	−	NA	+	+
**4)**	Excitable behavior [10], [13]–[21]	1B	+	+	+	−	NA	−	−
***Spatially uniform stimulus***									
**5)**	Freeze, cringe, spread [41]	2B	+	+	NA	−	NA	−	−
**6)**	Signaling events [32], [65], [66]	4B, C	−	+	NA	−	NA	−	−
**7)**	Adaptation [63], [64], [67]–[71]	4B, C	−	+	NA	+	NA	−	−
***Spatially graded stimulus***									
**8)**	Directional response/migration	4D–I		+	NA	+	+/−	+	+
**9)**	Biased patches [72]	6C	+	+	NA	−	NA	−	−
**10)**	Amplified response [42], [73]	4D–I	+	+	NA	+	+	−	−
**11)**	Simultaneous cues [42]	6A, B	−	+	NA	+	−	+	+
**12)**	Sensitivity adjustment [42], [68]–[70]	7D	−	+	NA	+	−	−	−
**13)**	Adjustment of polarity [74]	5A–F	−	−	NA	−	+	−	−
**14)**	Turning [24], [74], [75]	5A–F	+	−	NA	−	NA	−	+

A: Biased excitability with polarity models: [6], [9], [12].

B: LEGI-biased excitability without polarity models: [10]; other biased ENs [11], [76];

C: Excitability-only models: [7], [15], [17]; reviewed in Ref. [7].

D: LEGI models: [43], [77]–[79].

E: Polarization only: [45], [80]; others reviewed in Ref. [3], [4].

F: Stochastic with external bias models: [27], [81].

G: Stochastic with persistence [27], [30], [82] (*these are models that fit statistics, rather than signaling models).

The overall network topology has similarities with previously published models. Edelstein-Keshet and coworkers have proposed a number of models for cell polarity, motivated by the front-back appearance of Rho GTPases observed in neutrophils. Our model is similar to one of their proposed models (Model 4 in Ref. [45]). There, multiple positive feedbacks (or double negative feedback loops, for example Rac ⊣ Rho ⊣Cdc42→Rac, Rac→PIP_3_→Rac, Rac ⊣ Rho ⊣ PIP_3_→Rac) generate a bistable system. While no direct negative feedback loop is included, each of the GTPases is found in both active and inactive states, and so substrate depletion (the inactive states of the GTPases) can be considered as a negative feedback loop.

The combination of EN and Polarity modules is also comparable to a model proposed by Meinhardt [6] (who did not differentiate between the different processes) to explain chemotaxis. This model involved a positive feedback loop counterbalanced by two negative feedback loops – one local, the other global. Our combined Polarity-EN (excluding the LEGI mechanism) has two negative feedback paths, one that is local (*X*→*Y* ⊣ *X*), and the other global (*X*→*Y*→*W* ⊣ *P*→*X*). If we combine the two positive feedback loops of the excitable network (*X*→*X*) and the polarization model (*X*→*Y*→*Z*→*P*→*X*) then the topology of the models is similar. Neilson *et al.* carried out level set simulations of the Meinhardt model and generated results similar to ours for polarized cells, including pseudopod splitting, persistent random migration and turns in response to shallow gradients changing direction [12].

Models without a LEGI mechanism, or with only one positive feedback loop miss out on a number of important aspects of the overall response, however. These models do not adapt when given spatially uniform stimuli and cannot recreate the complex biphasic responses observed. Cells without an adaptation mechanism do not adjust sensitivity when the midpoint of the gradient is raised and hence perform less efficient chemotaxis (Fig. 7C,D). These simulations, however, show that adaptation is not absolutely required for chemotaxis. In fact, it is known that the response of migrating fibroblasts to uniform PDGF stimulation does not adapt, though these cells can only respond to gradients over a relatively narrow range of chemoattractant concentrations [46], as in our simulations of cells lacking the LEGI mechanism. In models with a single positive feedback loop, the simulated cells are always polarized. In reality, polarized and unpolarized cells can coexist in a population and cells can acquire increased polarity during a period of directed migration.

The ability of the polarized LEGI-BEN to simulate cell movement under a number of varying scenarios illustrates the relative complexity and sophistication of the chemotactic signaling machinery. Experiments have demonstrated that the pathways governing chemotaxis have considerable redundancy at the biochemical level [47]. Our simulations show a similar redundancy at a systems-level, as they demonstrate that directional migration can be achieved without a LEGI mechanism (Fig. 7C, D), or without polarity (Fig. 1F). However, both mechanisms improve efficiency. As argued above, the LEGI mechanism allows the cells to respond to chemoattractant gradients over a wide range of mid-point concentrations. The polarity mechanism enables cells to migrate persistently in the absence of chemoattractant gradients and allows them to use the small directional bias obtained from the gradient to focus most activity towards the source (Fig. 4E, G).

### Putative Biochemical Entities Associated with Model Elements

The modular framework of the polarized LEGI-BEN model gives rise to the entire spectrum of reported behaviors of cells but it is a conceptual model where individual biochemical entities are not assigned to specific model components or modules. An advantage of the modular approach is that, as additional data becomes available, the biochemical network within each module can be modeled in detail without altering the overall behavior of the other modules. Nevertheless, we can use several criteria to begin to assign various biochemical entities to the different modules (Table 2). First, the kinetic behaviors of certain biochemical and model components match under different conditions. Second, when levels of components and strengths of feedbacks within modules are varied, our simulated cells can “phenocopy” the behavior of various loss- and gain-of-function mutants.

**Table 2:** Putative model components.

Modules	Possible components
LEGI	Chemoattractant Receptors (GPCRs), G-proteins, Global Inhibitor (unknown)
Excitable network	Ras GTPases, Ras GEFs, Ras GAPs, PI3K, PTEN, PIP_3_, TorC2, PDKs, PKBs, PKB substrates, Rho family GTPases
Protrusion	Scar complex, Arp complex, formins, actin, actin-binding proteins
Polarity	Cytoskeletal proteins, membrane tension

We propose that the LEGI module incorporates the “upstream” components of the receptor signaling pathway (the chemoattractant-sensing GPCRs and associated G-proteins). Receptor-mediated G-protein dissociation is consistent with the local excitation process since during uniform or gradient stimulation they both rise rapidly and reach a steady-state level proportional to the level of receptor occupancy. Unfortunately, the biochemical identity of the global inhibition process that is expected to rise slowly and balance the persistent G-protein dissociation to bring about adaptation remains unknown. The receptors and G-proteins are not part of the excitable network since cells in the absence of chemoattractant or lacking G-protein function display excitability [18].

We propose that Ras and PI3K activity as well as other components traditionally viewed as elements of signal transduction pathways are part of the excitable network. Some of these, including Ras, PIP_3_, and Rac display excitable behavior such as wave propagation along the basal surface of the cell [10], [17], [19]–[21]. Furthermore, constitutive Ras activity and inhibition of PIP_3_ degradation cause excessive cytoskeletal activity and cellular extensions while inhibition of PI3K activity reduces this spontaneous activity [48]–[50]. We have also included many signal transduction components that either regulate, or are regulated by, Ras, PI3K, or Rac in this module. While biosensors are not available to directly test the premise, the participation of these components in propagating waves is expected since most of them behave coordinately with Ras and PI3K during uniform chemotactic stimulation. While many cytoskeletal proteins have also been shown to display excitable behavior, we have included these in the module that mediates protrusions [13]–[15], [18].

The polarity module is likely to include both cytoskeletal and signaling proteins and well as “polarity-specific” components. Cells with elevated levels of PIP_3_ or Ras activity or lacking myosin II appear to have decreased polarity [51]–[53]. However, it may be difficult to assign these components specifically to the polarity module since simulations in which the strengths of the negative feedback loops in either the polarization or excitable network modules are reduced lead to signaling levels and morphologies that are quite similar (Fig. 7A and Ref. [10]). Recently, it has been suggested that an activity akin to that achieved by *W* in the polarization module could be provided by membrane tension thus arguing for a role for cell mechanics [54], [55]. Cells with impaired dynacortin, a global actin linking protein, are softer and also form more pseudopods that are less aligned with the gradient, reminiscent of simulations in which *W* is reduced.

### Experimental Assessment of the Model

Here we suggest some possible experimental tests of our polarized LEGI-BEN model. 1) One assumption in the model is that the time-scale of adaptation (minutes) is longer than that of the excitable network (20–30 s). This can be tested by exposing cells to chemoattractant for several minutes, thus elevating the level of the global inhibitor in the LEGI module, and then removing the stimulus. Since excitation (*E*) is predicted to fall more rapidly than the inhibitor (*I*), the output of the LEGI module will transiently drop below its basal level. During this period of time, the spontaneous firing of the excitable network as well as its ability to be triggered by external stimulus will be decreased. 2) Currently the major evidence for excitability is observation of propagating waves. Further evidence of excitability could be obtained by testing whether cells generate all-or-none responses to supra-threshold stimuli, and whether they display a refractory period to repeated stimuli. According to our model, these hallmarks of excitable behavior should be largely independent of the actin cytoskeleton. 3) Treatment of cells with inhibitors of the cytoskeleton not only stops motility but also removes the polarity [42]. According to our model, without the mechanical or polarity module, a biased excitable network remains and activity is biased towards the gradient (Fig. 6C). We have recently found this prediction to be true when observing the dynamics of Ras and PI3K activity in cells treated with latrunculin in a steady gradient. 4) Our model hypothesizes that the persistence observed in unstimulated cells (item 1 in Table 1) is due to the same mechanism (the polarization module) that leads to polarized cells. One way to test this would be to track, in the absence of chemoattractant stimulus, the persistence of genetically modified cells that show poor polarity (e.g. cells lacking tsunami [56] or dynacortin [54]).

## Methods

### Signaling Network

We assume that the signaling network behaves as an excitable network [10]. It consists of two species (Fig. 1A). Component *X* acts as the activator: it is autocatalytic (it has strong positive feedback), and also activates the downstream component — we refer to this as the feedforward loop. The *Y* component provides negative feedback to *X*. A reaction-diffusion network, consisting of the following partial differential equations:
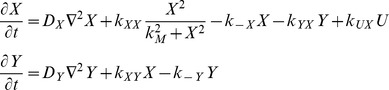
describes the evolution of the activities of these two species. Both components in this subsystem can diffuse spatially, with diffusion coefficients *D_X_* and *D_Y_*, respectively. The signal *U* is the input to the excitable system, which incorporates several components: a basal level of activation (*B*), a stochastic component (*N*), contribution from the LEGI response regulator (*R*, described below) and the polarization component (*P*, also described below). The contribution of each of these is additive:
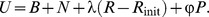
The stochastic component is modeled as zero mean, white noise process with variance 1. Note that, in this context, the external gradient and the internally developed polarity compete to direct cell motion [57], [58].

### Receptor Signaling: Local Excitation, Global Inhibition

The LEGI mechanism involves three interacting processes (Fig. 1E). An external signal, which represents the local level of receptor occupancy (*S*), drives two of them: a fast, local excitation (*E*), and a slow, global (diffusible) inhibitor (*I*). These two control a response regulator, which can be active (*R*) or not (*R_T_* – *R*), where we have assumed that the total concentration (*R_T_*) of the response regulator is constant. The system equations are given by:
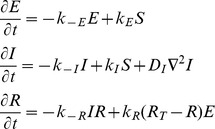



In the gradient simulations, the initial stimulus level is given by
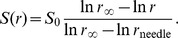
where *r* distance from each point on the cell boundary to the location of a hypothetical needle, which is either 10 µm (19% gradient) or 100 µm (6% gradient) away. This equation corresponds to the steady-state solution of the diffusion equation from a 5 µm needle in radial coordinates. The gradient is not updated as the cell moves to ensure that the gradient steepness is maintained. The gradient in our paper is defined as follows:
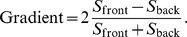



In simulations that tested the LEGI mechanism without inhibitor (Fig. 7B, C), the response regulator is described as follows:
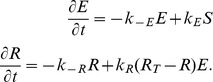



### Polarity Mechanism

The polarity mechanism is given by *P* = *Z−W*, where the individual components are also implemented as a local excitation, global inhibition mechanisms:
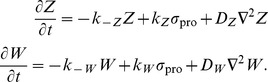
For simplicity, we let *D_w_* be sufficiently high that *W* is spatially independent. The polarization module is activated by signal *σ*_pro_, which represents actin polymerization and is proportional to *Y*.

### Model of Cellular Deformations

To determine the effect of the model activities on the shape of a cell we used a level set framework to simulate cell shape changes as previously described [22], [59]. In short, in the level set method (LSM) the cell is described as the zero-level set of a potential function φ(*x*,*t*), *x*∈**R^2^**. Initially, we use a signed distance function as the potential function, defined by:
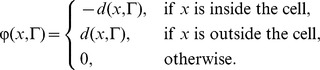
Here *d*(*x*,Γ) is the distance of position *x* to the cell boundary (initially a sphere of radius 5.1 µm). The evolution of the potential function is described by the Hamilton-Jacobi equation
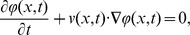
where *v*(*x*,*t*) describes the local velocity of the potential function. To obtain this velocity we apply different stresses on the cell and use a viscoelastic mechanical model of the cell to determine the local velocity. In our case we use:
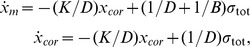
where σ_tot_ is the total stress applied on the cell, *x_m_* and *x_cor_* are the local displacements of the boundary and cortex, respectively, and *K*, *D* and *B* are viscoelastic components of the cell describing the elasticity (*K*) and viscosity (*D*) of the membrane, and the viscosity of the (*B*) of the cytoplasm. The velocity is given by *v* = d*x_m_*/d*t*.

The total net stress (σ_tot_) includes the vector sum of the stresses acting on the cell. This stress includes contributions from passive components, such as surface tension, σ_ten_ = γκ(*x*)***n***, where γ is the local cortical tension, κ is the local curvature, and ***n*** is a normal unit vector. Protrusive forces are proportional to the signal *Y* (using *X* leads to similar results) according to σ_pro_ = σ_0_ *Y*(*θ*)***n***, representing actin polymerization. The conversion factor between the activity *Y* and the force is 35 nN/µm^2^. Based on the typical maximum activity level for *Y* seen in the simulations (∼0.05 A.U.), this resulted in protrusive forces in the range of 1–3 nN/µm^2^, consistent with measured values of the maximum protrusive pressure due to actin polymerization (in the range of a few nN/µm^2^ [60], [61]). We also include a stress that acts to ensure surface area conservation, σ_vol_ = *k*_area_(*A*(*t*)-*A*_0_), where *A* is the surface area enclosed by the cell boundary either at time *t* or initially. Using these elements, we compute the total stress σ_tot_ = σ_pro_+σ_ten+_ σ_vol_ and use this to update the viscoelastic model parameters (*x_m_* and *x_cor_*) above.

### Model Implementation

The model and all simulations are implemented using Matlab (MathWorks, Natick, MA). Simulations were carried out in two steps. First, the PDEs for the signaling were solved around by representing the cell boundary as a one-dimensional system using periodic boundary conditions. This was discretized in space using 360 points. Spatial diffusion terms, which contain the second derivatives, are approximated by central differences in space; and by doing that, the partial differential equations are converted to ordinary differential equations. The solution of the stochastic differential equations was obtained using the SDE toolbox for Matlab [62]. The time step for simulation was set to 0.025 seconds. After solving the concentrations of all species (e.g. *X, Y, W, Z*), we compute the protrusive force using the concentration for *Y*, and use this protrusive force to update the potential function in the level set simulations, as described above. The potential function is solved on a Cartesian grid with spatial discretization of 19 points per µm. The assignment of *Y* activity levels to the protrusive force is done on a point-to-point pairing based on correspondence between angular positions relative to the cell centroid. The level set simulations were carried out using the Level Set Toolbox for Matlab using the first order forward Euler method [57].

### Parameters

All model parameters are found in Table 3. Parameters for the LEGI and EN components were used in our previously published model [10]. The parameters of viscoelastic model were obtained using by fitting experimental measurements of aspirated *Dictyostelium* cells using a micropipette, as previously reported [22], [59].

**Table 3:** Model parameters.

***Local excitation, global inhibition*** [10]			
*k_e_*	0.5 s^−1^	*k_-e_*	0.5 s^−1^
*k_i_*	0.1 s^−1^	*k_-i_*	0.1 s^−1^
*k_r_*	0.06 s^−1^	*k_-r_*	0.1 s^−1^
*R_T_*	2 A.U.	*D_I_*	1 µm^2^/s
*r_n_*	5 µm	*r_∞_*	1 m
*S_0_*	0.1 A.U. (0.2 in Fig. 7C)		
***Excitable signaling network*** [10]			
*D_X_*	1.6 µm^2^/s	*D_Y_*	3.8 µm^2^/s
*k_XX_*	2.5 s^−1^	*k_XY_*	0.019 s^−1^
*k_-X_*	2.3 s^−1^	*k_-Y_*	0.088 s^−1^
*k_M_*	0.32	*B*	−0.063
*k_YX_*	8.6 s^−1^	*R_init_*	1.25
*k_UX_*	0.8 s^−1^	*λ*	2
*φ*	2		
***Polarization*** * (this paper)*			
*k_z_*	0.023 s^−1^	*k_-z_*	0.015 s^−1^
*k_w_*	0.035 s^−1^	*k_-w_*	0.012 s^−1^
*D_z_*	4 µm^2^/s		
***Mechanical parameters*** [22], [59]			
*K*	0.098 nN/µm^3^	*D*	0.064 nN-s/µm^3^
*B*	6.09 nN-s/µm^3^	*γ*	1.00 nN/µm
σ*_0_*	35 nN/µm^2^		

To choose the parameters for the polarization model we used as a benchmark that the persistence of cells is in the order of two minutes. Since the equations driving the polarity module are linear, the appearance of a pseudopod (represented by a sudden increase in σ_pro_) leads to an increase in *Z* followed by an exponential decay with rate exp(−*k_−z_t*) (ignoring diffusion). Our nominal value for *k*_−*Z*_ is such that (1/*k*_−*Z*_) is approximately 1/66 seconds, which implies that the effect of that pseudopod is reduced to only e^−2^≈0.13 after two minutes. Note that during this time, more firings are possible, so that the effect of that initial pseudopod will likely be felt for longer periods. The time scale for the inhibitory element of the polarization module was chosen initially chosen in the same way. To arrive at the final values, we iterated in an *ad hoc* fashion, making sure that the activity of the excitable system (*X* and *Y*) showed some persistence in angle, but did not become locked in one position (sufficiently large feedback through the polarity module can lead to a bistable system). One way of measuring this is through the autocorrelation function *C*(*t*), measured at a fixed angles:
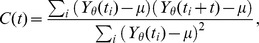
where μ is the mean level of signal. We computed this for unstimulated cells (where any persistence would come from the polarization module (Fig. S1) by choosing 10,000 angles and time points at random. Without the polarity module, the autocorrelation decays quickly (to less than 0.2) in approximately 30 seconds. With the nominal parameter values of the polarization module, there is a decay (the initial correlation can be accounted by the firings of the excitable system) but the autocorrelation plateaus at approximately 0.4. Increasing the timescale of the polarization component (by making the degradation slower can increase this plateau. Increasing the degradation constant of *Z* eliminates any long-term correlation.

We also carried out parameter sensitivity analysis on several components of the system. Previously, we have demonstrated that the LEGI mechanism is extremely robust to parameter variations. The parameters in the mechanical model were experimentally obtained [22], and tested previously. For this reason we focused our analysis on the polarization module and the excitable network. We had previously carried out sensitivity analysis on the latter, but because the polarity module acts in feedback with this, we included it in this analysis. This consisted of varying the nominal degradation/production rates and observing the spatial distribution of *X* and *Y*, which show similar patterns. Because these drive the mechanical model in open loop, we did not do extensive tests on morphology, being constrained by the computational burden of the level set simulations.

Our results show that small changes (±20%) in the rates controlling *X* and *Y* can have quite a strong effect on the excitable behavior (Fig. S2), as we noted previously [10]. These small differences in the rates of *Z* and *W* do not affect the spatial distribution of activity much or the peak levels of activity. To probe the robustness of the polarization module further, we also considered large (×10 or 1/10) changes in the parameters of the polarization module (Fig. S3). Changes of this size on the rates of *W* change the spatial distributions of *Y*, but by values smaller than the change in the parameter. For example, increasing the degradation of *W* tenfold only increases the peak activity level by approximately 60%; decreasing this rate to 1/10^th^ its nominal value decreases the peak level of activity to approximately half. Moreover, the spatial distribution is largely unaffected. Changing the rates of *Z* has the greatest effect.

### Chemotaxis Index

Chemotaxis index was computed using this following formula:
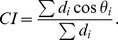
After application of the gradient the cell trajectory was sampled every 5 seconds: *P_i_*. The values *d_i_* are the distances from sample points *P_i_* and *P_i-1_*; *θ_i_* is the angle between the line connecting *P_i_* and *P_i-1_*, and direction of the gradient.

## Supporting Information

Figure S1 **Auto-correlation of Y activity.** Autocorrelation of the activity of *Y* for fixed angles θ under varying scenarios on the lifetime of the polarization module. Changes in the lifetime were obtained by varying the parameter *k_−Z_*. that specifies the degradation rate of *Z*.
(TIF)

Figure S2 **Parameter sensitivity.** These plots show the spatial distribution of *Y* under various parameter perturbations. Red line shows the mean level of activity for twenty, 900 s simulations. The shaded grey area represents one standard deviation.
(TIF)

Figure S3 **Parameter sensitivity for polarization.** These plots show the spatial distribution of *Y* under various parameter perturbations in the polarization module. Red line shows the mean level of activity for twenty, 900 s simulations. The shaded grey area represents one standard deviation.
(TIF)

Video S1 **Lack of persistence.** Simulation showing spontaneous protrusions in an unstimulated cell with no polarity (Fig. 1D).
(AVI)

Video S2 **Movement of an unpolarized cell in changing gradients.** Simulation of the LEGI-BEN module under changing 19% gradients. The initial 19% gradient, which points to the top was applied at 180 s. At 500 s, it was switched to point towards the bottom. This simulation corresponds to Fig. 1F, though it was rotated to fit the figure better.
(AVI)

Video S3 **Movement of polarized cells in the absence of a gradient.** This video shows the movement of five cells with the polarized LEGI-BEN modules, but no external gradient (as in Fig. 3F). Each cell was simulated individually, and the trajectories superimposed, so was possible for different cells to overlap in the movie.
(AVI)

Video S4 **Response of a polarized cell to a shift in the direction of a 6% gradient.** The initial 6% gradient was applied at 300 s and pointed to the right. At 900 s, the direction was shifted to point to the top. This video corresponds to the simulation in Fig. 5A. This simulation uses the polarization, LEGI and EN modules.
(AVI)

Video S5 **Response of an unpolarized cell to a shift in the direction of a 6% gradient.** The initial 6% gradient was applied at 300 s and pointed to the right. At 900 s, the direction was shifted to point to the top. This video corresponds to the simulation in Fig. 5C. This simulation uses the LEGI and EN modules.
(AVI)

Video S6 **Response of a polarized cell to a shift in the direction of a 19% gradient.** The initial 19% gradient was applied at 300 s and pointed to the right. At 900 s, the direction was shifted to point to the top. This video corresponds to the simulation in Fig. 5D. This simulation uses the polarization, LEGI and EN modules.
(AVI)

Video S7 **Development of polarity over a short exposure to a gradient.** This simulation uses the polarization, LEGI and EN modules. A 12% gradient is applied at the beginning of the simulation (pointing to the right) and redirected at 130 s (pointing to the top). This video corresponds to the simulation of Fig. 5E.
(AVI)

Video S8 **Development of polarity over a long exposure to a gradient.** This simulation uses the polarization, LEGI and EN modules. A 12% gradient is applied at the beginning of the simulation (pointing to the right) and redirected at 430 s (pointing to the top). This video corresponds to the simulation of Fig. 5F.
(AVI)

Video S9 **Response of cell to simultaneous gradients.** Competing 19% gradients were applied to the cell (forming a “V”-shape with the bottom of the “V” at the center of the cell.) This simulation uses the polarization, LEGI and EN modules. This video corresponds to the simulation of Fig. 6A.
(AVI)

Video S10 **Response of unpolarized cell to simultaneous gradients.** Competing 19% gradients were applied to the cell. This simulation uses the LEGI and EN modules. The red line marks the track of the cell centroid.
(AVI)

Video S11 **Response of immobilized cell to simultaneous gradients.** Competing 19% gradients were applied to the cell at 180 s. This simulation uses the LEGI and EN modules but sets protrusive stresses to zero. The video corresponds to Fig. 6B.
(AVI)

Video S12 **Response of immobilized cell to single 19% gradient.** A single 19% gradient, pointing to the right, was applied to the cell at 180 s. This simulation uses the LEGI and EN modules but sets protrusive stresses to zero. The video corresponds to Fig. 6C.
(AVI)

Video S13 **Response of cells with varying polarization modules loop strengths altered.** The cells are responding to a 19% gradient pointing to the right. This video corresponds to Fig. 7A.
(AVI)

Video S14 **Simulation of cells lacking adaptation.** This simulation shows the response of the cell to a 19% gradient pointing to the right. The inhibitor level of the LEGI mechanism has been set constant, so the cell cannot adapt or adjust sensitivity. This video corresponds to Fig. 7B.
(AVI)

Video S15 **Simulation of cells lacking adaptation in higher midpoint concentration.** This simulation shows the response of the cell to a 19% gradient pointing to the right. The inhibitor level of the LEGI mechanism has been set constant, so the cell cannot adapt or adjust sensitivity. The midpoint chemoattractant concentration has been increased. This video corresponds to Fig. 7C.
(AVI)
