# Supplementary figures and images for: Interaction of Motility, Directional Sensing, and Polarity Modules Recreates the Behaviors of Chemotaxing Cells
Source: PLoS Comput Biol. 2013 Jul 4;9(7):e1003122. doi: 10.1371/journal.pcbi.1003122 (PMC3701696; doi:10.1371/journal.pcbi.1003122)

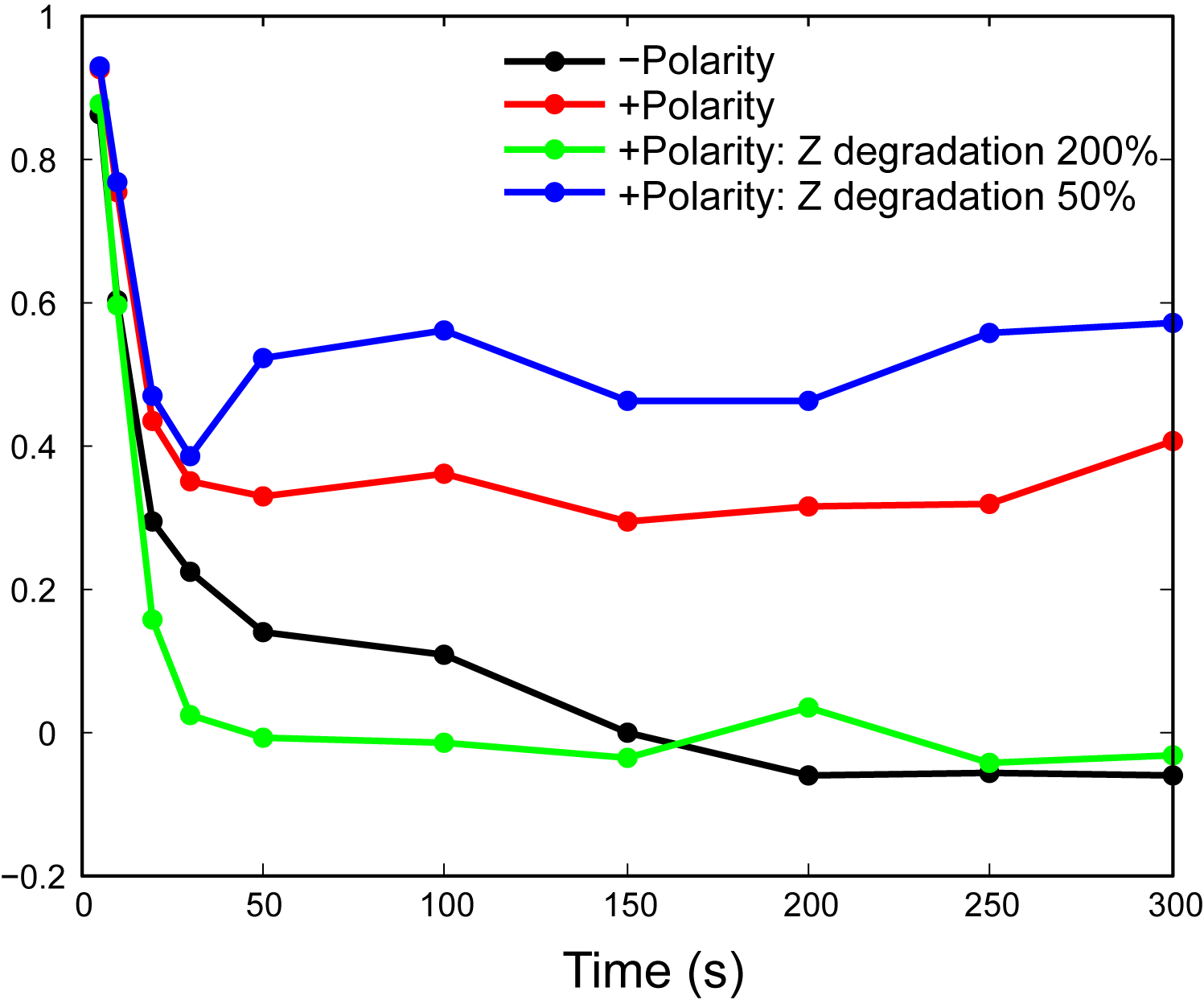

Supplement: Figure S1 — Auto-correlation of Y activity. Autocorrelation of the activity of Y for fixed angles θ under varying scenarios on the lifetime of the polarization module. Changes in the lifetime were obtained by varying the parameter k−Z. that specifies the degradation rate of Z. (TIF) [file pcbi.1003122.s001.tif]

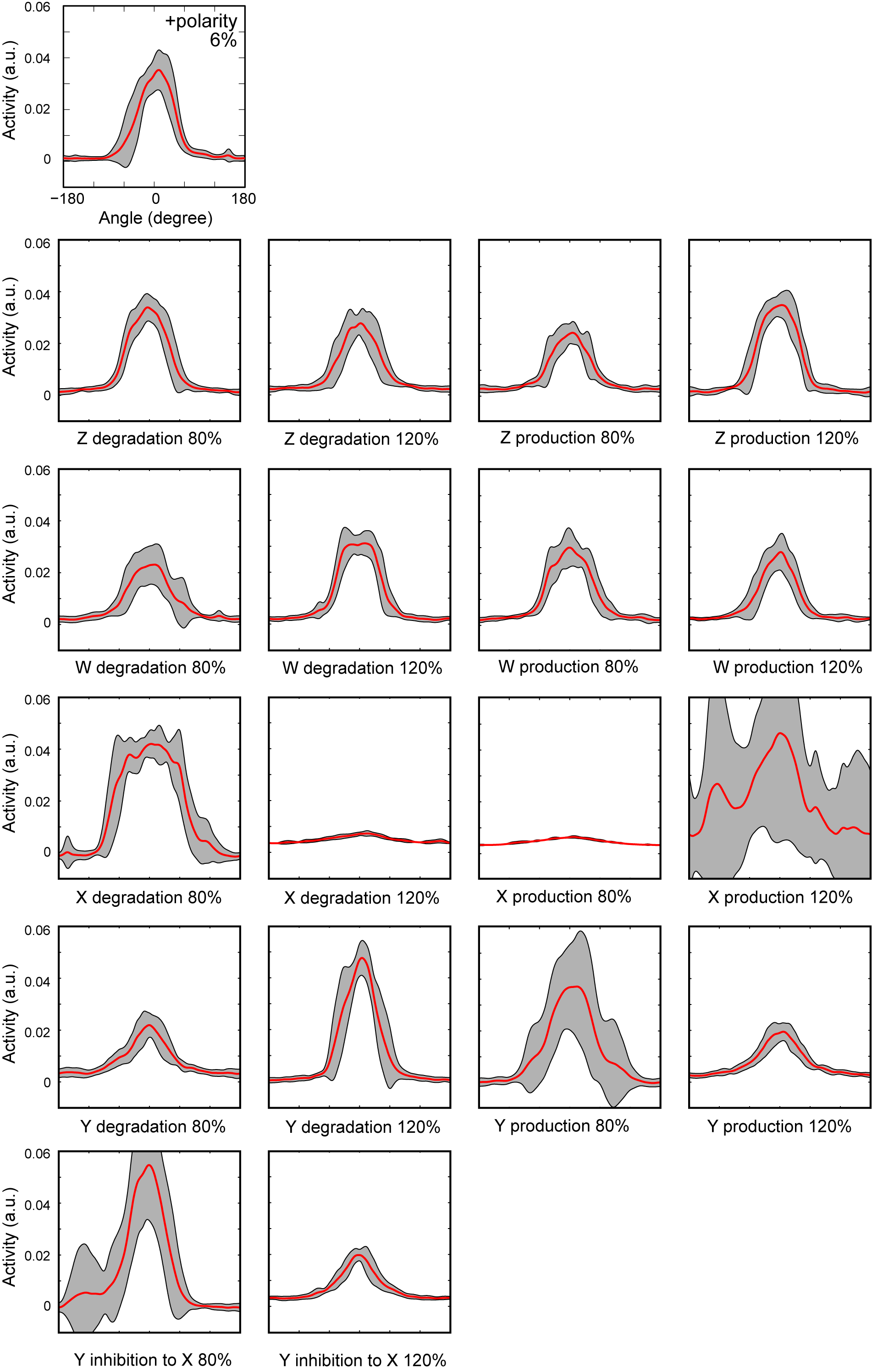

Supplement: Figure S2 — Parameter sensitivity. These plots show the spatial distribution of Y under various parameter perturbations. Red line shows the mean level of activity for twenty, 900 s simulations. The shaded grey area represents one standard deviation. (TIF) [file pcbi.1003122.s002.tif]

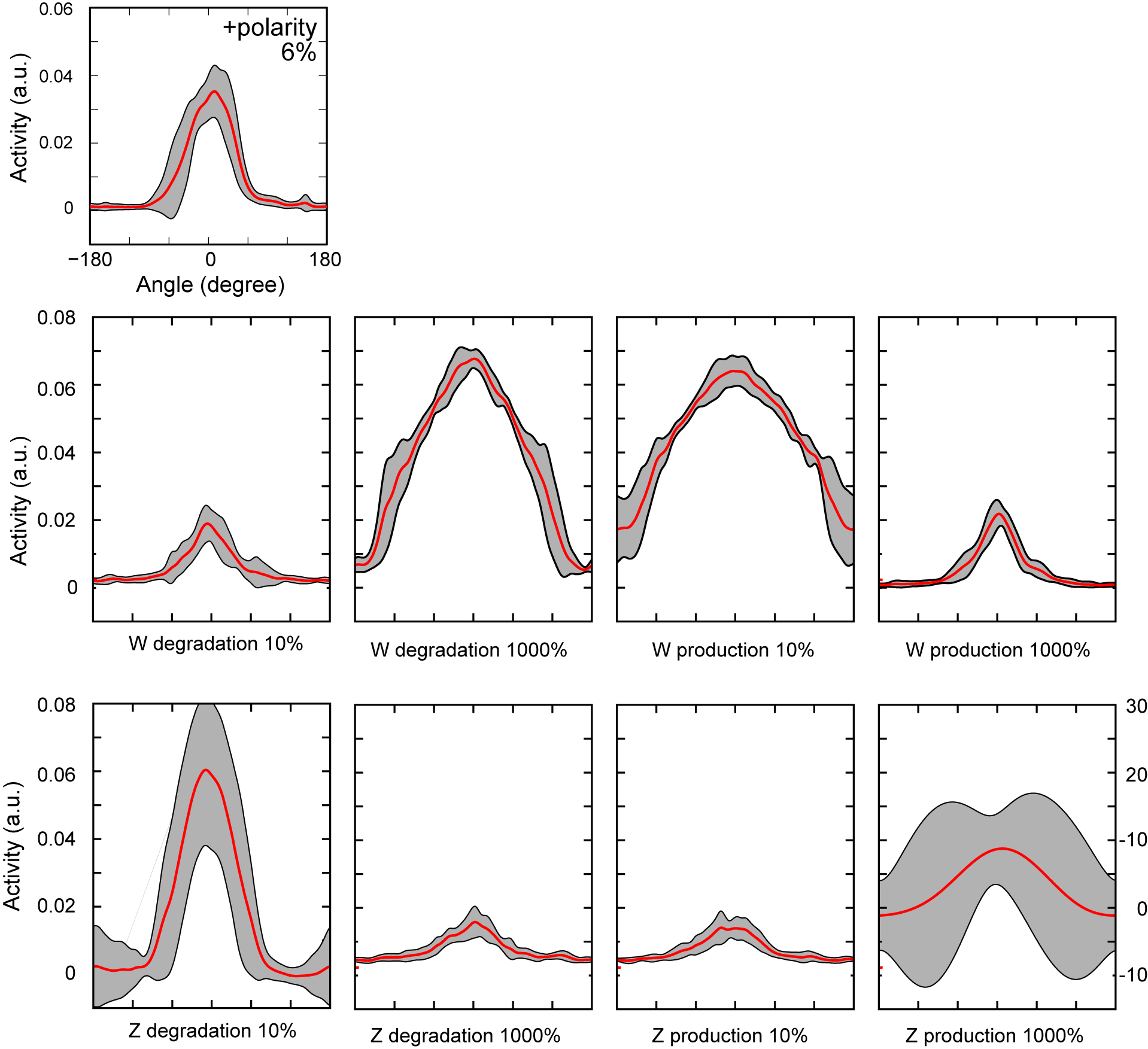

Supplement: Figure S3 — Parameter sensitivity for polarization. These plots show the spatial distribution of Y under various parameter perturbations in the polarization module. Red line shows the mean level of activity for twenty, 900 s simulations. The shaded grey area represents one standard deviation. (TIF) [file pcbi.1003122.s003.tif]
